# Effects of Physicochemical Variables of Superficial Waters on the Abundance of the North African Freshwater Crab *Potamon algeriense* (Bott, 1967)

**DOI:** 10.1155/2021/6669919

**Published:** 2021-03-16

**Authors:** Soufiane Fadlaoui, Ouahid El Asri, Mustapha Bouterfas, Mohammed Melhaoui

**Affiliations:** ^1^Mohamed First University, Faculty of Sciences, Department of Biology, Laboratory of Water, Environment, and Sustainable Development, Oujda, Morocco; ^2^Microbiology and Biotechnology Laboratory, Faculty of Sciences, Ibn Zohr University, Agadir, Morocco

## Abstract

The large number of pollutants discharged into the aquatic environment may influence the physicochemical and biological qualities of the aquatic ecosystem. This study discloses the global quality of the surface waters and the effect of physicochemical variables on the abundance of the African freshwater crab *Potamon algeriense* inhabiting Zegzel watercourse, a mountain stream in the northeast of Morocco. Physicochemical variables including streamflow, water temperature, pH, dissolved oxygen, suspended matter, chloride, calcium, magnesium, ammonia, nitrite, nitrate, orthophosphate, and organic matter were evaluated monthly for one year (October 2017–September 2018). The evaluation of the physicochemical quality showed that the waters of all the stations studied are between the excellent and good quality classes concerning all the physicochemical variables and highlights also two variations with a tendency towards degradation, one spatial from upstream to downstream and the other seasonal from the wet to the dry period. The abundance of crabs was recorded to exhibit a positive correlation with dissolved oxygen, calcium (*N* = 44) (*p* < 0.01), and magnesium. However, a negative correlation has been noticed for streamflow (*N* = 1) (*p* < 0.01), water temperature, pH, suspended matter, chloride, ammonia, nitrite, nitrate, orthophosphate, and organic matter. The results obtained reveal that besides the biotic variables, the distribution of *P. algeriense* also depends on these specific environmental variables.

## 1. Introduction

Freshwater quality deserves special attention because it is a vital resource, and it has recently been much altered and seriously threatened by human activities such as physicochemical and biological pollution caused by runoff from agricultural and urban areas and the overexploitation due to the anarchic water diversion.

Pollution of aquatic bodies has caused several environmental problems, such as the disappearance of biodiversity and aquatic ecosystems [1]. To overcome poor water quality stress, aquatic organisms can alter their metabolism, which results in severe changes in metabolic cycles involving biochemical constituents of tissues [2]. Crustaceans are highly sensitive to pollution [2, 3], and their abundance and existence depend on particular environmental variables such as pH and dissolved oxygen [4, 5].

Studies on the determination of correlation between abiotic factors and crab populations seem to be imperative to evaluate the effects of extremes of physicochemical variables on species and provide information regarding the optimum conditions under which crabs can reproduce and maintain the population.

The freshwater crab *P. algeriense* (Bott, 1967) is one of the largest freshwater macroinvertebrates, as its carapace width can reach more than 60 mm [6]. This species belongs to the Potamidae family, and it is found exclusively in three countries: Morocco, Algeria, and Tunisia [7]. In Morocco, *P. algeriense* has been reported in freshwater including rivers, ponds, lakes, dams, running water streams from the north in the watershed of Oued Laou near Chefchaouen, from the northeast in the watershed of Moulouya, and from the Middle Atlas in the watershed of Oued Oum Er-Rbia near Khenifra [6].

Despite the wide distribution of *P. algeriense*, its occurrence drastically declined during the last two decades due to human impacts (sewage, industrial waste, and agricultural pesticides) [6].

Compared to marine brachyurans, which have been the focus of many studies [8–12], few studies have been carried out on the abundance and population structure of freshwater decapods [13]. *P. algeriense* makes no exception; in fact, except for a few studies on the biology of this species [6, 14, 15], only one study has been performed concerning the ecological aspect of this species [16].

The present study aims to evaluate the global physicochemical quality of the surface water and to investigate the effect of these variables on the distribution of populations of the freshwater crab *P. algeriense* along Zegzel watercourse, a mountain stream in the northeast of Morocco. For this purpose, we looked at correlations between abiotic factors and crab population abundance to evaluate the effects of physicochemical variables on this species and provide information regarding the optimum conditions under which crabs can reproduce and maintain a population.

## 2. Materials and Methods

### 2.1. Study Area

**Figure 1:**
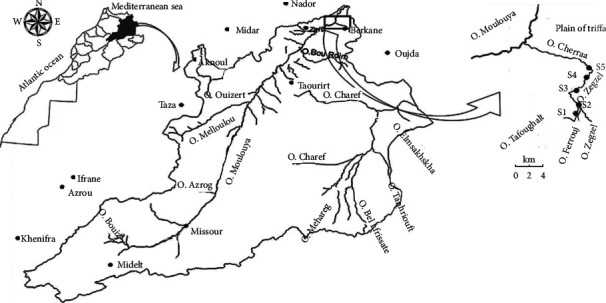
Localization of the study area, Zegzel watercourse, Morocco.

The present study was performed in the Zegzel watercourse (34° 50′ 29.4″ N, 2° 21′ 19.8″ W), a tributary of the lower Moulouya River in the northeast of Morocco. In this investigation, five stations were selected (Figure 1).

- Station 1 (upstream) (34°50′18.93″ N 2°21′53.87″ W) is considered as a virgin station, no dysfunctions were observed, and the local inhabitants depend on this station for their daily drinking water
- Station 2 (34°50′24.63″ N 2°21′21.74″ W) faces various bad practices such as washing the dishes (use of detergents) and discharge of solid and liquid wastes by tourists
- Station 3 (34°50′50.36″ N 2°21′34.62″ W) waters are used by the local inhabitants for swimming and agriculture purposes. Also, this station is frequented by tourists due to the presence of cafes on its banks.
- Station 4 (34°51′52.74″ N 2°22′09.10″ W) is considered as a virgin station due to its difficult accessibility. No kind of dysfunctions was detected.
- Station 5 (downstream) (34°52′22.32″ N 2°21′49.08″ W) is considered as the most polluted station of Zegzel watercourse. Various and serious sources of pollution have been noticed such as cloth and car washing, livestock drinking, anarchic irrigation of agricultural plots, and massive discharge of solid and liquid wastes.

**Figure 2:**
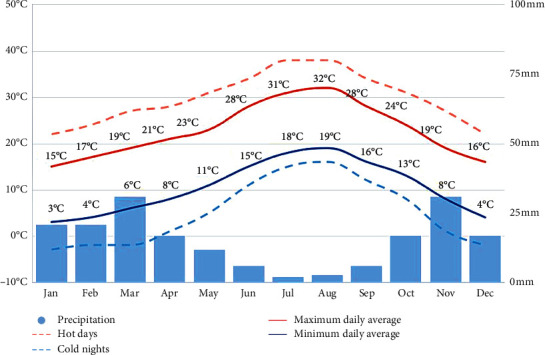
Climodiagram of the study area, Zegzel watercourse, Morocco.

Concerning the climate, the study area is located under a typically Mediterranean climate given to its position on the southwestern edge of the Mediterranean Sea. Rainfall is very irregular, and there is no average year of precipitations but rather a succession of dry and wet years (Figure 2).

### 2.2. Water Sampling

For the analysis of the physicochemical variables, surface water samples were collected monthly over one year (October 2017–September 2018) from the five sampling stations where animals were collected. The samples were taken by hand from the middle of the watercourse in clean polyethylene bottles, kept immediately in a cooler at (4°C), and then transported as soon as possible to the laboratory.

The water samples were analyzed at room temperature and atmospheric pressure for suspended matter (SM), chloride (Cl^−^), calcium (Ca^2+^), magnesium (Mg^2+^), ammonia (NH_4_^+^), nitrite (NO_2_^−^), nitrate (NO_3_^−^), orthophosphate (PO_4_^3−^), and oxidability (Oxid) by adopting the standard protocol of Rodier [17] (Table 1).

Besides laboratory analysis, some variables were determined at the sampling sites to avoid the introduction of bias. In fact, at each sampling station, water temperature (T°) and dissolved oxygen (DO) were measured using a portable oximeter (Orion Star A123), while the pH was measured using a pH meter (Jenco 6175).

Streamflow, average speed, wet section, average water width, and depth were also measured on the site.

### 2.3. Calculation of the Physicochemical Water Quality Index

The physicochemical water quality index used in our study is a system developed by the Directorate of Water Resources and Planning in Morocco [18]. This system has the advantage of being accurate and complete. It is based on the notion of the weighted quality index which is calculated for each variable.

The formula for calculating the weighted index is as follows:

(1) $$\begin{array}{c} \text{WIpa}=\text{Li}+\left[\frac{\left(\text{Hi}-\text{Li}\right)}{\left(\text{Hb}-\text{Lb}\right)}\right]\times \left(\text{Hb}-\text{Pa}\right), \end{array}$$

where WIpa is the weighted index of the variable analyzed, Li is the lower index, Hi is the higher index, Lb is the lower bound, Hb is the higher bound, and Pa is the variable analyzed.

The values of the intervals set by the new grids for assessing water quality are transformed into unitless values ranging from 0 for very bad quality to 100 for excellent quality [18] (Table 2).

The general surface water quality grid sets five quality classes, each defined by a set of threshold values that the various variables must not exceed [18, 19].

### 2.4. Crabs Collection

Crabs were collected monthly over one year (October 2017–September 2018) by excavating burrows and searching under blocks. Crabs could be caught by plunging an arm into the burrow; then, they were put in a half filled bucket of water. Generally, each station took an average of 4 hours of excavating by one person. Catch per unit effort (C.P.U.E.) was determined following the formula of Maunder et al. [20]:

(2) $$\begin{array}{c} \text{C}.\text{P}.\text{U}.\text{E}=\frac{\text{Total catch}}{\text{Total no}.\text{ of effort}}. \end{array}$$

All crabs were released, on the same day of their catch, in their natural habitat after collecting necessary data.

### 2.5. Statistical Processing of Data

All the statistical analyses were performed by SPSS 23 software. Spearman's correlation test was used to determine the relationship between physicochemical variables and the existence and abundance of *P. algeriense*. The choice of this statistical test was based on a prior study of the normality of the variables before their transformation.

## 3. Results and Discussion

Monthly data on C.P.U.E have been calculated for the five studied stations (Table 3). The values of C.P.U.E never remained constant, but they follow a fluctuating profile over the year and the stations. It is evident that higher values of C.P.U.E have been registered in stations 1 and 4; however, lower ones were observed in stations 2 and 3. C.P.U.E values also follow periodic fluctuations. In fact, higher values have been recorded in dry season (from August to January) with a maximum value (C.P.U.E = 41.25); however, lower ones were reported during wet season (from February to July) with a minimum value (C.P.U.E = 3).

The values of the hydromorphometric variables obtained from the five study stations are shown in Table 4.

Station S1 is the deepest of all stations with an average depth of 0.48 ± 0.31 m. Nevertheless, station S2 is the shallowest with an estimated mean depth of 0.33 ± 0.11 m. With an average width of 9.02 ± 0.57 m, station S4 is the widest, while station S1 is the narrowest with width of 3.45 ± 0.14 m. The highest speed values were recorded at station S3 (1.06 ± 1.08 m/s). However, the lowest average speed was recorded at station S1 (0.26 ± 0.24 m/s). The mean streamflow was generally highly variable from the station to the other with values ranging from 0.04 ± 0.02 m^3^/s at station S1 to 0.36 ± 0.44 m^3^/s at station S3.

During the study period, 13 physicochemical variables were evaluated to disclose their effects in the repartition and the abundance of *P. algeriense* in the Zegzel watercourse. Besides global climate change, which presents a major factor controlling the past and future distribution of freshwater crab biodiversity, abiotic variables may also contribute to this distribution [21–23]. The physicochemical variables are strongly crucial environmental factors, as it directly influences diversity, oxygen consumption, growth rates, metabolism, molting frequencies, hormones, and survival of crustaceans [24, 25].

In order to reveal the influence of physicochemical variables on crab abundance, a correlation has been performed. The values of correlation are given in Table 5. Few positive correlations have been remarked between crab abundance and certain variables such as dissolved oxygen, calcium, and magnesium. However, negative correlations have been obtained for the rest of the variables (streamflow, temperature, and pH).

**Figure 3:**
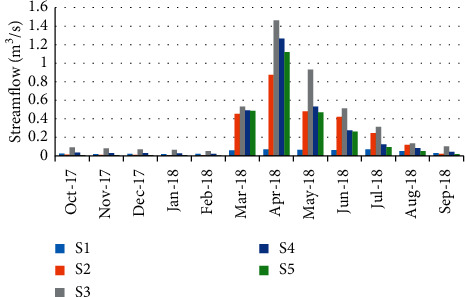
Spatial-temporal variation of streamflow in the Zegzel watercourse.

The streamflow values were varied from 0.005 in February (station 5) to 1.462 m^3^/s during April (station 3) (Figure 3). The abundance of the crab population has shown a negative correlation (*r* = −0.986) with the streamflow (Table 5).

Streamflow is an essential factor for the ecology of running waters, and it is closely linked to the climate of the region and the nature of the watershed (soil permeability, vegetation cover, and anarchic irrigation). The spectacular difference in streamflow between the period March and June and the other months of the year in the study area is the result of the heavy rainfall through this period; however, low streamflow in summer may be due to the scarce precipitations, the high evaporation rate, and the anarchic derivation of water to irrigate agricultural parcels located on the banks of the watercourse. The strong flow could destroy crab habitat, therefore the disappearance of the crabs (pers. obs), which explains the high negative correlation.

**Figure 4:**
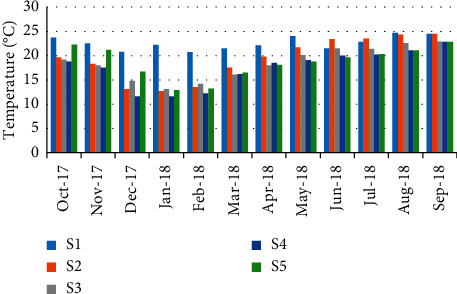
Spatial-temporal variation of water temperature in the Zegzel watercourse.

Concerning water temperature, minimum values were recorded in December and January (11.6°C) (station 4); however, maximum temperature (24.7°C) was recorded in August (station 1) (Figure 4). The results showed a highly negative correlation (*r* = −0.881) between water temperature and the abundance of crabs (Table 5).

Temperature is an essential and important factor because of its direct or indirect influence on several ecological variables. The monthly variations of water temperature in the Zegzel watercourse showed that it follows a seasonal pattern, which was fluctuated according to the prevailing atmospheric temperatures. Temperature is a limiting factor in the aquatic environment. It disturbs the metabolic activities, growth, oxygen consumption, reproduction, molting frequency, survival, existence, and migratory behaviors of crustaceans [26]. The temperature affects blood clotting times [27], reduces the metabolic rate [28], increases blood cell numbers [29], induces a change in osmotic pressure of the hemolymph [30], affects the antibacterial activity by hemocytes [31], and the phagocytosis mechanism [32]. All these effects may explain the high negative correlation recorded between this variable and the crab abundance.

**Figure 5:**
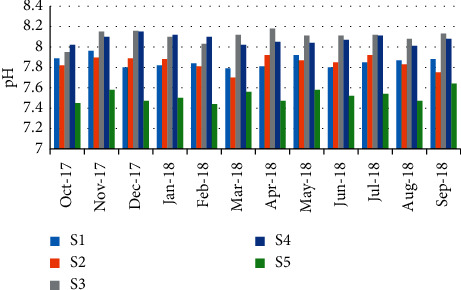
Spatial-temporal variation of pH in the Zegzel watercourse.

During the assessment period, minimum pH (7.44) was recorded in February; however, maximum pH (8.14) was recorded in April (station 3) (Figure 5). The results showed a negative correlation (*r* = −0.853) between pH and the abundance of *P. algeriense* (Table 5).

pH is an important factor in the determination of the productivity of an ecosystem. The pH of water in different stations of the Zegzel watercourse was observed to be more basic. This basicity may be essentially due to the geological nature of the study area (limestone) and the photosynthetic activity [33]. pH has a great influence on the existence and the abundance of crabs, and it may be due to its effect on the immunity system of crabs by stopping the activity of the phenoloxidase enzyme, which helps to protect against microbial infection in arthropods. pH influences also the metabolism, physiology, and maturation process of crustaceans [34].

**Figure 6:**
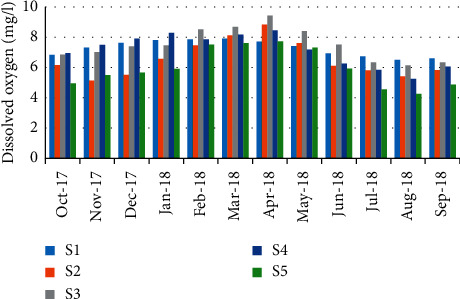
Spatial-temporal variation of dissolved oxygen in the Zegzel watercourse.

The minimum concentration of dissolved oxygen 4.26 mg/L was recorded in August (station 5); however, a maximum of 9.43 mg/L was recorded in April (station 3) (Figure 6). The results show a high positive correlation (*r* = 0.941) between water oxygenation and the abundance of crabs (Table 5).

Dissolved oxygen is one of the most important environmental factors influencing the distribution of biodiversity. Variation of the dissolved oxygen in the study area may be due to various factors such as temperature, dissolved salt concentration (dissolved oxygen decreased due to increased temperature and salinity of water [35]), photosynthetic activity of plants, decomposition of organic matter, and respiration by micro and macroorganisms [36]. Dissolved oxygen concentrations plainly affect the behavior of crustaceans [37, 38] and life strategies based on oxygen consumption and energy content [39]. Oxygen consumption has been reported in several previous studies in different species of decapods [2, 40–42]. Low concentrations of dissolved oxygen levels may induce stress by creating a kind of anaerobic metabolism particularly of glucose in these crabs, which could result in lactate production and acidosis [43–46] and could also reduce growth and molting frequencies [47], change in osmotic pressure of the hemolymph [30], reduction of metabolic rate [28], and finally cause mortality [48].

**Figure 7:**
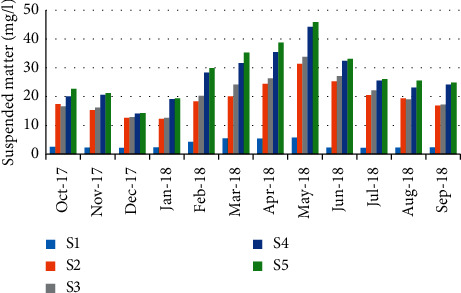
Spatial-temporal variation of suspended matter in the Zegzel watercourse.

The concentrations of suspended matter varied from 2.2 mg/L during December (station 1) to 45.9 mg/L in May (station 5) (Figure 7). The results showed a negative correlation (*r* = −0.623) between suspended matter values and the abundance of crabs (Table 5).

Suspended matter includes any mineral or organic material that does not dissolve in water. It causes the degradation of the optical water quality by reducing water clarity and decreasing sunlight available to support the mechanism of photosynthesis [49]. Higher concentrations of suspended matter have been recorded from March to June, which may be the result of the sudden hydrological events (floods), whose load is the suspended matter that can be attributed to an intense erosion of the watershed, aggravated by vegetable washing in the watercourse. High amounts of suspended matter induce serious problems for the aquatic life, from phytoplankton to crustaceans, mainly when the particles have a small diameter which may transport many dangerous substances that are harmful or lethal and may clog crab gills. In freshwater ecosystems, these small particles constitute a food source for filter feeders, which are part of the food chain, leading to the biomagnification phenomenon of chemical pollutants in crustaceans. The suspended matter may also cause serious damage affecting the biotope of these species which can blanket the streambed consequently, destroying the crab habitat [50].

**Figure 8:**
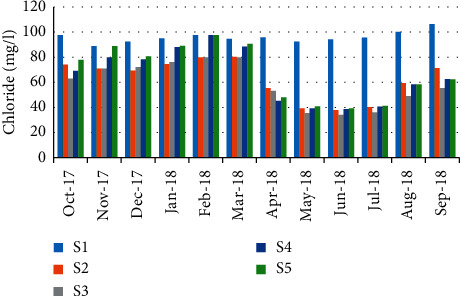
Spatial-temporal variation of chloride in the Zegzel watercourse.

For chloride, the minimum concentration was recorded in June 33.78 mg/L (station 4); however, the maximum value (106.34 mg/L) was recorded in September (station 1) (Figure 8). The correlation between chloride values and the abundance of *P. algeriense* showed a negative pattern (*r* = −0.321) (Table 5).

Chloride is one of the main anions of natural water influencing the existence of fauna and flora. Chloride concentrations recorded in the Zegzel watercourse may be due to the agricultural wastes inducing fertilizers and effluents of human and animal wastes. High chloride concentrations result in an increase in salinity, which may reduce the crab abundance. Furthermore, large amounts of chloride in lotic and lentic ecosystems may also affect the gills of freshwater fish as well as freshwater crabs [51]. All these harmful effects may explain the negative correlation observed between the abundance of this species and this variable.

**Figure 9:**
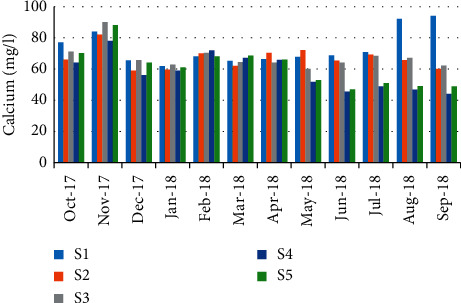
Spatial-temporal variation of calcium in the Zegzel watercourse.

Concerning calcium concentrations, the minimum value was recorded in September (41.7 mg/L) (station 4); however, the maximum was recorded in November (95.54 mg/L) (station 4) (Figure 9). A highly positive correlation (*r* = 0.933) has been observed between calcium values and the abundance of crabs (Table 5).

**Figure 10:**
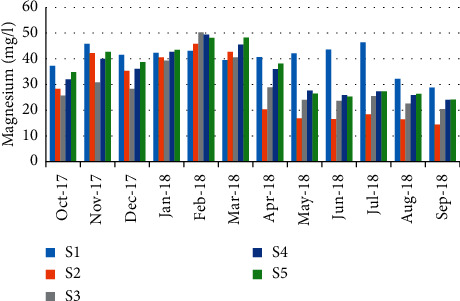
Spatial-temporal variation of magnesium in the Zegzel watercourse.

For magnesium, minimum concentration was recorded on September 14.4 mg/L (station 2); however, a maximum 50.33 mg/L was recorded in February (station 2) (Figure 10). The values show a high positive correlation (*r* = 0.769) between magnesium concentrations and the abundance of crabs (Table 5).

The presence of the calcium and magnesium cations results in the hardness of water. High concentrations of calcium and magnesium in the Zegzel watercourse are attributed to the interactions between water and geological fragmentation of the area (limestone). The hardness of water could also be derived from CO_2_ released in bacterial action from the soil through in percolating water. Increased concentrations observed in the summer were mainly due to rising temperature, thereby increasing the solubility of these cations (Ca^2+^ and Mg^2+^) [52]. Calcium and magnesium are very vital components for crabs, as it is required during calcification (formation of the exoskeleton) [53]. However, deficiency in the concentrations of these cations causes prolonged periods of the soft exoskeleton (delay in the process of calcification) and makes freshwater crustaceans more vulnerable to predation and consequently limits the distribution and the existence of crabs [54]. Such effects may explain the high positive correlations recorded between the existence of this species and these cations.

**Figure 11:**
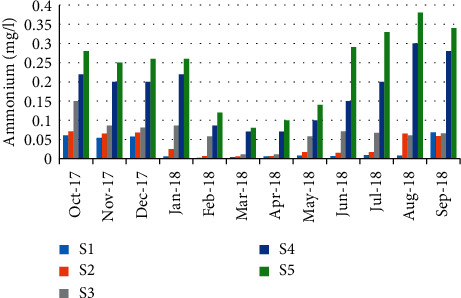
Spatial-temporal variation of ammonia in the Zegzel watercourse.

For ammonia concentrations, the minimum value (0.002 mg/L) was recorded during February (station 1); however, the maximum was during August (0.38 mg/L) (station 5) (Figure 11). A negative correlation (*r* = −0.685) has been recorded between ammonia results and the abundance of crabs (Table 5).

Ammonia is one of the elements of the complex nitrogen cycle in its primitive state. The higher concentrations in summer could be partially due to the death and subsequent decomposition of phytoplankton and to the excretion of ammonia by plankton [55]. In contrast, lower concentrations of ammonia were observed from March to June due to water dilution (rainy season). Ammonia is very toxic to aquatic life, which induces serious problems for crustaceans, such as inducing damage in various organs, affecting nutrients and proteins that they use for survival and growth. Ammonia could also have lethal and sublethal effects on crustaceans [56].

**Figure 12:**
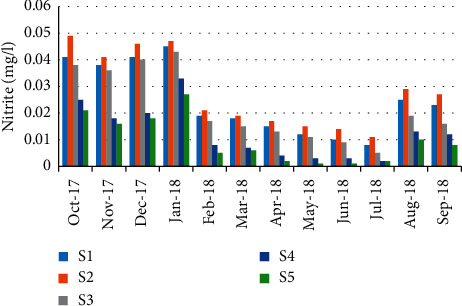
Spatial-temporal variation of nitrite in the Zegzel watercourse.

Nitrite concentrations varied from 0.001 mg/L during May and June (station 5) and 0.049 mg/L during October (station 2) (Figure 12). The results showed a negative correlation (*r* = −0.547) between nitrite concentration and the abundance of crabs (Table 5).

Nitrite is probably the most important environmental variable. They constitute a transient form of nitrogen between nitrate and ammonia ions. The higher nitrite values recorded in the Zegzel watercourse during late summer, autumn, and the winter beginning could be due to the increased planktonic excretion, oxidation of ammonia, and reduction of nitrate and due to bacterial decomposition of plankton detritus present in the environment [57, 58]. Furthermore, the denitrification and air interaction exchange of chemicals could also be responsible for these increased values [59]. However, low nitrite concentrations recorded from March and June may be due to water dilution (rainy season). High values of nitrites affect directly the reproduction, metabolic activities, behavior, growth, and create abnormal conditions of crustacean inhabitants.

**Figure 13:**
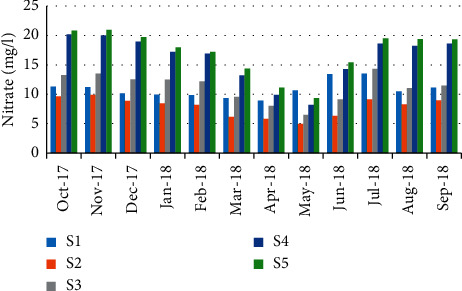
Spatial-temporal variation of nitrate in the Zegzel watercourse.

Nitrate results were fluctuated from 4.96 mg/L in May (station 2) to 20.95 mg/L during November (station 5) (Figure 13). Like the case of nitrites, a negative correlation (*r* = -0.597) has been noticed between nitrate concentrations and the abundance of crabs (Table 5).

Nitrate is the final stage of nitrogen oxidation and represents the highest nitrogen form of oxidation present in water. The low concentration of nitrate observed from March to June might be due to the heavy rainfall (water dilution); however, the maximum concentration of nitrate recorded during the summer could be due to direct discharges of organic and agricultural wastes [60]. Low streamflow and evaporation during this period may also rise these concentrations [61]. An excessive amount of nitrate inputs may result in degradation of water quality due to the noxious effect of algal blooms, which causes oxygen deficiency and habitat loss leading to the disappearance of crabs. However, a reasonable amount of nitrate may favor the rational growth of algae, which presents one of the food materials in the diet composition of the *P. algeriense* (unpublished results).

**Figure 14:**
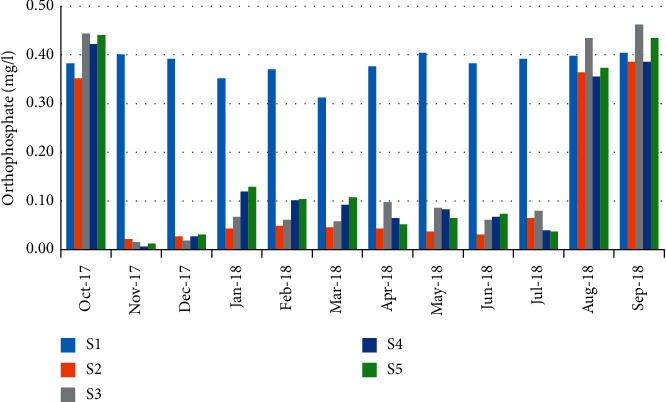
Spatial-temporal variation of orthophosphate in the Zegzel watercourse.

The orthophosphate concentrations were varied from 0.01 mg/L in November and December (stations 4 and 5) to 0.46 mg/L during September (station 3) (Figure 14). Like the case of nitrogen compounds, a negative correlation (*r* = −0.612) has been observed between orthophosphate values and the abundance of crabs (Table 5).

Phosphate is one of the most important nutrients that can be found in different oxidized forms in water. Low orthophosphate concentrations were observed during the period between March and June due to the heavy rainfall which causes water dilution. However, high concentrations recorded during late summer and the beginning of autumn might have resulted from sewage and fertilizer disposal from the agricultural lands. Orthophosphate and the other nitrogen compounds are the main factors of eutrophication, which results in algae blooms that cloud the water and block the sunlight from reaching important underwater flora that furnishes the building block for the aquatic food web and shelter for juveniles to adult crustaceans. High changes in the concentrations of orthophosphate entering the water may also cause disturbance of ecological balances and affect the quality of freshwater ecosystem life. Furthermore, excess nutrients could also induce serious problems in the cellular arrangement of the hepatopancreas and damage gonads of freshwater crabs [62, 63].

**Figure 15:**
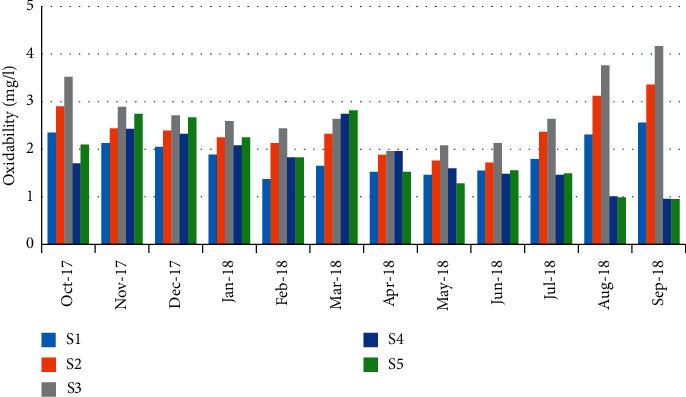
Spatial-temporal variation of oxidability matter in the Zegzel watercourse.

The organic matter values were varied from 0.95 mg/L during the early autumn in September (station 5) to 4.16 mg/L during September (station 3) (Figure 15). The values showed a highly negative correlation (*r* = −0.796) between organic matter and the abundance of crabs (Table 5).

The organic and oxidizable materials represent all the substances whose presence causes the consumption of the dissolved oxygen in the water. The low organic matter concentrations recorded during the period from March to June indicate effective assimilation of organic load due to the large concentrations of oxygen. In contrast, maximum concentration values were observed during summer and the beginning of autumn due to the direct discharges of organic wastes and low dissolved oxygen concentrations, which affect the assimilative capacity ensured by the activity of aerobic bacteria. Great concentrations of organic matter could drastically reduce the available dissolved oxygen, and consequently, crabs and other aquatic species are suffocated.

After the calculation of the weighted quality index (WIpa) using the results of physicochemical analyses and the standard values of the Moroccan norm, the water quality class is determined for the 60 samples relating to the 5 sampling stations (Table 6).

The evaluation of the physicochemical quality showed that the waters of all the stations studied are between the excellent and good quality classes concerning all the physicochemical variables. Overall, the results obtained highlight two variations with a tendency towards degradation, one spatial from upstream to downstream and the other temporal seasonal from the wet to the dry period (Table 6).

The upstream-downstream variation along the Zegzel watercourse is relatively clear. The deterioration of water quality from upstream to downstream of the rivers is mainly linked to liquid and/or solid discharges and the impact of agricultural activities [64, 65]. At Zegzel watercourse, it results in a mineral and organic load of anthropic origin from tourist and agricultural activities. On the other hand, the quality of the water is improving as a result of the phenomenon of dilution of pollutants by numerous sources (Merkida and Bourbah) which feed this watercourse and due to the floods which the region knows during the wet period (February–May). Talhaoui et al. [66] also concluded in a similar work on the Moulouya River that pollution increases from upstream to downstream.

The seasonal variation is also clear; it shows an increase in water quality from February to July. On the other hand, there is a slight evolution towards degradation from August to January (Table 6). The increase in the degree of pollution is linked to the decrease in the flow of the Zegzel watercourse during the dry period and the canalization of water for the irrigation of agricultural plots. Similar works have shown that river pollution is relatively higher during the dry season than during the wet season [66–68].

Concerning the effect of physicochemical variables on the abundance of *P. algeriense*, our results are in accordance with other similar works which have shown that the existence and distribution of freshwater crabs depend on these abiotic factors. Indeed, Sharma et al. [69] observed that the freshwater crab *Paratelphusa masoniana*, inhabiting a tributary of River Chenab, Jammu, exhibit a negative correlation with water temperature, depth, pH, and chloride; however, there is a positive correlation with dissolved oxygen, calcium, magnesium, and bicarbonate.

Sakhare and Kamble [63] also discussed the influence of physicochemical variables over the reproduction of the freshwater crab *Barytelphusa cunicularis*. Altered physicochemical variables influence the gametogenic mechanism of *Barytelphusa cunicularis*, and testicular cells showed hypertrophy which reduced content of gametogenic cells leading to infertility. Sakhare and Kamble [62] also showed that cellular arrangement of *Barytelphusa cunicularis* was disturbed by water pollution, suggesting that such physicochemical changes would lead to progressive loss of basic biological functions of the hepatopancreas.

## 4. Conclusion

The present study focused on the calculation of the global quality of the surface waters of the Zegzel watercourse and the influence of physicochemical variables on the population of the freshwater crab *P. algeriense*.

The assessment of physicochemical water quality has shown that all the stations surveyed are among the excellent and good quality class throughout all physicochemical variables. Also, the global water quality index showed a seasonal and spatial variation from upstream to downstream. Thus, the level of deterioration becomes relatively high during the dry summer period and would be linked to the decrease in the flows of the Zegzel watercourse.

It can also be concluded that besides the biotic variables comprising reproduction, food availability, and predation, the abundance and distribution of the freshwater crab *P. algeriense* may also be under the influence of various environmental factors. The abundance of crabs has shown a positive correlation with dissolved oxygen, calcium, and magnesium. However, a negative correlation has been recorded for streamflow, water temperature, pH, suspended matter, chloride, ammonia, nitrite, nitrate, orthophosphate, and organic matter.

## Figures and Tables

**Table 1:** Analysis methods of the physicochemical variables.

Variables analyzed	Unit	Methods of analysis
Suspended matter	mg/l	Filtration on filter paper (Whatman)
Oxidability	mg/l	Determination by the potassium permanganate method
Chloride	mg/l	Volumetric method using mercuric nitrate
Calcium	mg/l	EDTA titrimetric method
Magnesium	mg/l	EDTA titrimetric method
Nitrate	mg/l	Determination by the spectrophotometric method with sodium salicylate reagent
Nitrite	mg/l	Determination by the spectrophotometric method with diazotization reagent
Ammonia	mg/l	Determination by the spectrophotometric method with sodium phenol nitroprusside reagent
Orthophosphates	mg/l	Determination by the spectrophotometric method with ammonium molybdate reagent

**Table 2:** Extract from the general grid of physicochemical quality used for the classification of surface water [18].

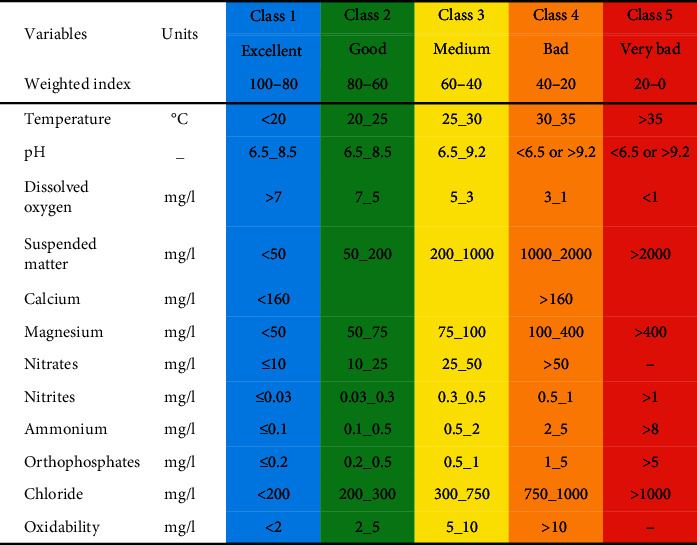

**Table 3:** Calculated values of catch per unit effort of *P. algeriense* of 5 stations along Zegzel watercourse.

	Station 1	Station 2	Station 3	Station 4	Station 5
Wet period (February–July)	19.5	2.75	3	17.25	13
Dry period (August–January)	24.25	16.5	10.75	41.25	19

**Table 4:** Mean values and standard deviation of hydromorphological variables measured at the five sampling stations during the study period.

Stations	Variables			
	Width (m)	Depth (m)	Speed (m/s)	Streamflow (m^3^/s)
S1	3.45 ± 0.14	0.48 ± 0.31	0.26 ± 0.24	0.04 ± 0.02
S2	4.57 ± 0.26	0.33 ± 0.11	0.81 ± 0.91	0.22 ± 0.28
S3	6.24 ± 0.17	0.39 ± 0.15	1.06 ± 1.08	0.36 ± 0.44
S4	9.02 ± 0.57	0.42 ± 0.05	0.62 ± 0.63	0.25 ± 0.37
S5	5.62 ± 0.67	0.41 ± 0.09	0.43 ± 0.53	0.21 ± 0.34

**Table 5:** Spearman's correlations between crab abundance and environmental variables.

Variables	*R* values	Significance
Streamflow	−0.986	*p* < 0.01
Temperature	−0.881	*p* < 0.01
pH	−0.853	*p* < 0.01
Dissolved oxygen	0.941	*p* < 0.01
Suspended matter	−0.623	*p* < 0.05
Chloride	−0.321	*p* < 0.05
Calcium	0.933	*p* < 0.01
Magnesium	0.769	*p* < 0.05
Ammonia	−0.685	*p* < 0.01
Nitrites	−0.547	*p* < 0.05
Nitrate	−0.597	*p* < 0.05
Orthophosphate	−0.612	*p* < 0.05
Organic matter	−0.796	*p* < 0.01

**Table 6:** Weighted quality index values and surface water quality class of the Zegzel watercourse.

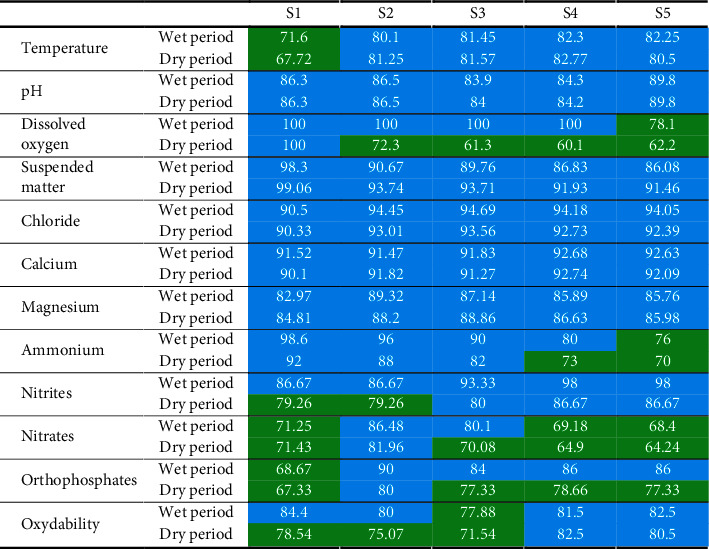

## Data Availability

The data used to support the findings of this study are available from the corresponding author upon request.
